# Combined Gaussian Mixture Model and Pathfinder Algorithm for Data Clustering

**DOI:** 10.3390/e25060946

**Published:** 2023-06-16

**Authors:** Huajuan Huang, Zepeng Liao, Xiuxi Wei, Yongquan Zhou

**Affiliations:** 1 College of Artificial Intelligence, Guangxi Minzu University, Nanning 530006, China; 2 Guangxi Key Laboratory of Hybrid Computation and IC Design Analysis, Nanning 530006, China

**Keywords:** clustering, Gaussian Mixture Models, metaheuristic algorithm, pathfinder algorithm

## Abstract

Data clustering is one of the most influential branches of machine learning and data analysis, and Gaussian Mixture Models (GMMs) are frequently adopted in data clustering due to their ease of implementation. However, there are certain limitations to this approach that need to be acknowledged. GMMs need to determine the cluster numbers manually, and they may fail to extract the information within the dataset during initialization. To address these issues, a new clustering algorithm called PFA-GMM has been proposed. PFA-GMM is based on GMMs and the Pathfinder algorithm (PFA), and it aims to overcome the shortcomings of GMMs. The algorithm automatically determines the optimal number of clusters based on the dataset. Subsequently, PFA-GMM considers the clustering problem as a global optimization problem for getting trapped in local convergence during initialization. Finally, we conducted a comparative study of our proposed clustering algorithm against other well-known clustering algorithms using both synthetic and real-world datasets. The results of our experiments indicate that PFA-GMM outperformed the competing approaches.

## 1. Introduction

Clustering is a fundamental tool in machine learning that facilitates the extraction of underlying similarities from data by grouping similar data points into clusters based on their features. Clustering has been widely applied in diverse domains, ranging from network analysis and business to marketing, education, data science, and medical diagnosis [1]. Clustering, a fundamental technique in data analysis, involves grouping similar objects or data points together based on some similarity metric. We explore the two primary approaches to clustering: partitional clustering and hierarchical clustering [2]. Each method has its own advantages and disadvantages, and the appropriate method should be chosen based on the characteristics of the data and the problem at hand [3]. Clustering can be conceptualized as an optimization problem that aims to categorize all data into distinct groups, where the inter-cluster distance is maximized and the intra-cluster distance is minimized. This process is executed using various algorithms that may produce different clustering outcomes [4]. Gaussian Mixture Models (GMMs) are a powerful statistical tool that enables the analysis and clustering of complex datasets that do not conform to a single distribution. One issue with GMMs is that they require the number of components to be specified in advance. Choosing an inappropriate number of components can lead to overfitting or underfitting, which can compromise the accuracy of the model. It means manual selection of cluster numbers is necessary for GMMs. GMMs have the potential to be inadequate in capturing complex patterns within data during initialization, which may result in suboptimal performance. To enhance the performance of GMMs, it is crucial to explore other techniques and algorithms that can capture complex patterns more efficiently [5].

The shortcomings of GMMs can be summarized as follows:

- To perform clustering using GMMs, one needs to manually configure the cluster numbers. However, this task can be challenging and can significantly impact the outcome of the clustering process;
- The initialization phase of GMMs may encounter difficulties in capturing complex structures within the data and become trapped in local convergence, leading to suboptimal clustering performance.

The Pathfinder algorithm (PFA) is included as a solution for the deficiency of GMMs, due to its strong global search ability and simple yet effective concept, enabling it to converge to the optimal solution in a relatively small number of iterations [6]. We proposed a novel clustering algorithm called PFA-GMM, which combines GMMs and PFA to address the aforementioned issues.

- To address the issue of Gaussian Mixture Models potentially getting stuck in local convergence during initialization, we introduced the powerful global searching PFA for clustering analysis. PFA identifies the optimal solution during initialization, thereby avoiding the local convergence trap;
- In addressing the issue of manual selection of cluster centers by GMMs, we employed the Davies–Bouldin Index as a fitness function for PFA, allowing for the automatic selection of cluster numbers.

The paper is structured as follows: Section 2 provides a summary of the related work relevant to this study. Section 3 presents the theoretical basis and some related concepts. In Section 4, we propose a new clustering algorithm based on GMMs and PFA called PFA-GMM. Section 5 analyzes the experimental results on different datasets. Finally, we provide a summary of our work in Section 6.

## 2. Related Work

Finite mixture modeling is a statistical technique that examines whether model parameters vary over unmeasured groups of individuals. The goal is to estimate the parameters of a mixture distribution, which is a probability distribution that results from the combination of two or more probability distributions [7,8].

Gaussian Mixture Models (GMMs) are a well-known method for modeling probability distributions of continuous variables. They are flexible and powerful tools for a wide range of applications, such as image processing, speech recognition, and data clustering. Chen et al. [9] establish the information-theoretic threshold for the separation of cluster centers, which ensures the exact retrieval of cluster labels in a K-component Gaussian mixture model with equal cluster sizes. Qu et al. [10] proposed a novel GMMs-based algorithm for anomaly detection in Hyperspectral Images with the aim of improving the detection accuracy of anomaly pixels and an effective GMMs-based weighting approach for fusing the extracted anomaly result. Fu et al. [11] introduce a novel embedded feature selection approach for GMMs by incorporating a relevancy index. The relevancy index is a metric that quantifies the probability of assigning a data point to a specific clustering group, thereby facilitating feature selection. Patel et al. [12] compare the cluster representativeness of K-Means and GMMs for heterogeneity in resource usage of cloud workloads. Experimental results suggest that GMMs are superior to K-Means when it comes to clustering with distinct usage boundaries. Although GMMs require more computational time, they are more effective in fine-grained workload characterization and analysis.

Projection pursuit is a statistical technique used for exploratory data analysis, information visualization, and feature selection [13]. It is a broader framework that has a form of an additive model of the derived features rather than the inputs themselves [14].

Nature-inspired algorithms, also known as metaheuristic algorithms, have gained widespread popularity in the field of engineering due to their remarkable ability to solve complex problems. These algorithms seek to find the optimal solution by balancing exploitation and exploration in the search space [15]. Compared to traditional clustering, which is susceptible to local convergence and initialization, metaheuristic algorithms have a higher probability of achieving the optimal global solution [16]. Yapici et al. [6] proposed the Pathfinder algorithm in 2019, a swarm intelligence algorithm inspired by the leadership of animal hunting behavior. Animals living in groups often make decisions based on the social hierarchy among members and may need to make decisions with or without a leader. Varaprasad et al. [17] utilized the Pathfinder algorithm to optimize the allocation and integration of a solar photovoltaic system. The Pathfinder algorithm was applied to determine the optimal configuration of Interline-Photovoltaic (I-PV) systems among multiple feeders to enhance the performance and resilience of the distribution system operation and control while maintaining various operational and radiality constraints. Oladipo et al. [18] employed an innovative approach to optimize the control efficiency of electrical systems. Specifically, they combined the flower pollinated algorithm (FPA) with the Pathfinder algorithm (PFA) to achieve maximum efficiency. By leveraging the PFA’s ability to ensure the search for optimal solutions, the authors were able to successfully exploit the full potential of their algorithmic approach. Tang et al. [19] have devised a novel approach by combining two algorithms, namely the teaching–learning-based algorithm (TLBO) and the Pathfinder algorithm (PFA), to enhance the exploration and mining abilities of the original algorithm. TLBO facilitates the teaching phase by considering the pathfinder of PFA as a teacher, thereby increasing the exploration ability of the algorithm. On the other hand, it allows the followers of PFA to perform the learning phase in TLBO and apply it to the mathematical model of PFA to enhance the mining ability of the algorithm.

## 3. Theoretical Background

We provide a comprehensive introduction to the Pathfinder algorithm and Gaussian Mixture Models.

### 3.1. Pathfinder Algorithm

The searching, exploiting, and hunting abilities of animal swarms have always been a focus of interest for many scientists. All behaviors in a swarm are carried out on the basis of the common action of all individuals [20]. The hunting skills of animal swarms have long captivated the attention of scientists. Mimicking the behavior of social movement within animal species, the Pathfinder algorithm simulates the characteristics of searching for prey or feeding areas under the guidance of a leader within animal herds. There is a leader corresponding to multiple members of the population, and the members of the population follow the leader according to the location of a neighbor and the behavior of the leader [21]. The algorithm begins by randomly initializing the positions of herd members. Afterward, the fitness of each individual is calculated, and the position of the individual with the best fitness is chosen as the pathfinder to be followed [22]. The pathfinder updates the location through Equation (1), and the members update the location through Equation (2).

(1) $$x_{i}^{K+1}=x_{i}^{K}+R_{1}\times \left(x_{j}^{K}-x_{i}^{K}\right)+R_{2}\times \left(x_{p}^{K}-x_{i}^{K}\right)+\varepsilon ,\;\;\;i\geq 2$$

(2) $$x_{p}^{K+1}=2r_{3}\left(x_{p}^{K}-x_{p}^{K-1}\right)+A$$

where *K* represents the current iteration, $x_{i}^{K}$ represents the position vector of ith member, $x_{p}^{K}$ represents the position of the pathfinder, $x_{j}^{K}$ is the position vector of jth member, $R_{1}$ is equal to $\alpha r_{1}$, and $R_{2}$ is equal to $\beta r_{2}$, where $r_{1}$, $r_{2}$, $r_{3}$ are random variables uniformly generated in the range of [0, 1], and α and β are randomly selected in the range of [1, 2] over the course of iterations. ε and A represent members and pathfinder random movement, respectively, are given by Equations (3) and (4)

(3) $$A=u_{2}\times e^{\frac{-2K}{K_{m\alpha x}}}$$

(4) $$\varepsilon =\left(1-\frac{K}{K_{max}}\right)\cdot u_{1}\cdot D_{ij},\;\;\;D_{ij}=\left|x_{i}-x_{j}\right|$$

where $u_{1}$ and $u_{2}$ variables are set in the range [−1, 1], and members can also move to their previous positions. $D_{ij}$ is the distance between two members; $K_{max}$ represents the maximum iteration. Locating the global optimum of optimization problems is a significant challenge. Therefore, we assume that the best solution detected thus far is the global optimum and accept it as the food area or hunt area to be exploited by the herd. In the Pathfinding algorithm, the objective is to locate the optimal food source or hunting ground, also known as the global optimum. During each iteration, the pathfinder’s position is designated as the current optimal location, and the other members of the group converge toward it. The pseudo-code is given as follows (Algorithm 1).
**Algorithm 1:** Pseudo-code of the Pathfinder algorithm
1.  Initialize the population
2.  Calculate the fitness of initial population
3.  Find the Pathfinder
4.  While *K* < maximum number of iterations
5.           *α* and *β* = random number in [1, 2]
6.           Update the position of Pathfinder using Equation (1)
7.           If new Pathfinder is better than old
8.                   Update Pathfinder
9.            End
10.          For *i* = 2 to maximum number of populations
11.                  Update positions of members using Equation (2)
12.          End
13.          Calculate new fitness of members
14.          Find the best fitness
15.          If best fitness < fitness of Pathfinder
16.                  Pathfinder = best member
17.                  Fitness = best fitness
18.          End
19.          For *i* = 2 to maximum number of populations
20.                  If new fitness of member(*i*) < fitness of member(*i*)
21.                          Update members
22.                  End
23.          End
24.          Generate new *A* and ε
25. End

### 3.2. Gaussian Mixture Models and Expectation Maximization

Gaussian Mixture Models (GMMs) are one of many unsupervised clustering techniques that are typically trained using an Expectation–Maximization (EM) algorithm to maximize the likelihood [23]. In GMMs, each cluster is considered an independent Gaussian distribution, also known as a normal distribution. This approach is used to assign data points to clusters based on the probability distribution [24], and the Gaussian distribution is defined as Equation (5):

(5) $$N\left(X|\mu ,\Sigma \right)=\frac{1}{{\left(2\pi \right)}^{\frac{D}{2}}\sqrt{\left|\Sigma \right|}}exp\left\{-\frac{{\left(X-\mu \right)}^{T}\Sigma^{-1}\left(X-\mu \right)}{2}\right\}$$

where μ is a *D* dimensional mean vector, and Σ is a covariance matrix with *D* × *D* shapes. The Gaussian distribution is a probability distribution that is defined by its mean and standard deviation. While the unimodal Gaussian distribution is inadequate to represent multiple density regions found in multimodal datasets, they can be effectively modeled using GMMs. The GMMs are defined as Equation (6):

(6) $$P\left(X\right)=\sum_{k=1}^{K}\pi_{k}N\left(X|\mu_{k},\Sigma_{k}\right)$$

where *K* is the number of Gaussian mixture distribution, and $\pi_{k}$ is the weight of *k*th Gaussian distribution, where the sum of $\pi_{k}$ is one, $\mu_{k}$ denotes the mean of *k*th Gaussian distribution, and $\Sigma_{k}$ represents the covariance matrix of the *k*th Gaussian distribution. The Gaussian mixture distribution comprises a convex combination of Gaussian distributions, providing significantly more flexibility to model complex densities than a simple Gaussian distribution. If we seek to obtain the maximum likelihood estimation, we need to derive the log-likelihood function as the following Equation (7).

(7) $$\ln p\left(X|\pi ,\mu ,\Sigma \right)=\sum_{n=1}^{N}\ln\left\{\sum_{k=1}^{K}\pi_{k}N\left(x_{n}|\mu_{k},\Sigma_{k}\right)\right\}$$

we cannot obtain a closed-form solution through maximum likelihood estimation. However, we can simplify it by introducing a latent variable, which turns out to be the EM algorithm for GMM. The *E*-step and *M*-step for GMM are displayed as Equations (8) and (9), respectively.

(8) $$Q_{i}\left(z_{i}^{k}\right)=p\left(z_{i}^{k}|x_{i};\theta \right)=\frac{\pi_{k}N\left(x_{i};\pi_{k},\Sigma_{k}\right)}{\sum_{k=1}^{K}\pi_{k}N\left(x_{i};\pi_{k},\Sigma_{k}\right)}$$

(9) $$\pi_{k}=\frac{\sum_{i=1}^{M}Q_{i}\left(z_{i}^{k}\right)}{M},\mu_{k}=\frac{\sum_{i=1}^{M}x_{i}Q_{i}\left(z_{i}^{k}\right)}{\sum_{i=1}^{M}Q_{i}\left(z_{i}^{k}\right)},\Sigma_{k}=\frac{\sum_{i=1}^{M}\left(x_{i}-\mu_{k}\right){\left(x_{i}-\mu_{k}\right)}^{T}Q_{i}\left(z_{i}^{k}\right)}{\sum_{i=1}^{M}Q_{i}\left(z_{i}^{k}\right)}$$

The fundamental principle of the EM algorithm is to revise one parameter at a time while keeping the others fixed. The algorithm proceeds iteratively by calculating the *E*-step and *M*-step until convergence is reached.

## 4. Pathfinder Algorithm Based on GMMs for Data Clustering

In this paper, we propose PFA-GMM, a new clustering algorithm that integrates the Pathfinder algorithm with GMMs for data clustering. As GMMs are highly susceptible to initialization, we introduce the Pathfinder algorithm to mitigate this issue. To measure the intra-cluster compactness and inter-cluster separation, we employ the Davies–Bouldin (DB) index as the fitness function [25].

### 4.1. Internal Validation Criteria

We can categorize criteria for evaluating the quality of clusters into two types: internal validation and external validation. These criteria assess the effectiveness of clustering and help validate the resulting clusters [26]. The fundamental distinctions between internal validation and external validation of cluster analysis are primarily contingent upon the utilization of external information. In practical applications, such as clustering labels, it may be unavailable. Consequently, internal validation, which relies on the information within the dataset, is often the sole recourse for evaluating clusters in the absence of external information [27]. Internal validation is a method used to evaluate the effectiveness of clustering algorithms by assessing inter-cluster separation and intra-cluster compactness.

- Compactness: It measures the proximity of intra-cluster data. Variance has been utilized for measuring compactness in some methods, with lower variance representing better compactness. Similarity also has been applied to measure compactness, such as pairwise distance;
- Separation: The inter-cluster data distinction is measured using a similarity metric to determine the distance between sets of clusters. This distance is used to evaluate separation, such as the pairwise distance of intra-cluster data points and the distance between cluster centroids.

Davies–Bouldin (DB) index: The DB index assesses the average inter-cluster similarity between any two clusters and their nearest counterparts. The Davies–Bouldin Index is calculated as the average similarity of each cluster with a cluster most similar to it. The clustering results are enhanced by minimizing this index; the lower the Davies–Bouldin Index, the better the clusters are separated, and the better the result of the clustering performed. This reduction in the DB value highlights the distinctiveness of each cluster, thereby producing an optimal clustering outcome. The Davies–Bouldin Index is defined as follows:

(10) $$DB=\frac{1}{c}\sum_{i=1}^{c}\underset{i\neq j}{\max}\left\{\frac{d\left(x_{i}\right)+d\left(x_{j}\right)}{d\left(c_{i},c_{j}\right)}\right\}$$

where *c* represents the number of clusters; *i* and *j* are cluster labels; $d\left(x_{i}\right)$ and $d\left(x_{j}\right)$ denote the distance of all the entities in clusters *i* and *j*; $d\left(c_{i},c_{j}\right)$ represents the distance between cluster centroids.

### 4.2. PFA-GMM

PFA-GMM comprises three components. First, the proposed method selects cluster centers from the dataset by calculating fitness values to determine the number of resulting clusters, and PFA-GMM introduces the Pathfinder algorithm for clustering analysis. The population is initialized based on the candidate cluster centers. The PFA’s global search ability is leveraged to discover multiple optimal solutions. These optimal solutions are then utilized as the initial cluster centers. By doing so, the PFA-GMM algorithm successfully accomplishes the task of automatically selecting the cluster centers, thus eliminating the subjectivity that arises from manual selection processes.

Second, the candidate solutions are iteratively applied to data clustering using the EM algorithm. By updating the population through the Pathfinder algorithm, it is possible to obtain the optimal solution during initialization and avoid getting trapped in local convergence.

Finally, the Pathfinder algorithm dynamically updates an optimal population and pathfinder until the termination condition is achieved. Additionally, searching for an optimal solution for data clustering. According to the above description, the main frame of PFA-GMM is presented below (Algorithm 2):
**Algorithm 2:** Pseudo-code of PFA-GMM**Input: The set of data points**$X=\{x_{1},x_{2},\cdots ,x_{n}\}$**, Maximum iterations, Population numbers** **Output: The optimal clustering result**
1.  Initialize the population
2.  Select data points randomly and determine the number of clusters C
3.  Generate the initial population and applying through GMM
4.  Calculate the fitness function according to EM
5.  Choose population with the best fitness value as Pathfinder
6.  While *K* < maximum number of iterations
7.       For *i* = 1 to maximum number of populations
8.              Update positions of members using Equation (2)
9.              Calculate fitness value of members through EM
10.      End
11.      If best fitness < fitness of Pathfinder
12.             Pathfinder = best member
13.             Fitness = best fitness
14.      End
15.      *α* and *β* = random number in [1, 2]
16.      Generate new A and ε
17.      Update the position of Pathfinder using Equation (1)
18.      If new Pathfinder is better than old
19.             Update Pathfinder
20.             Calculate fitness value
21.      End
22. Assign data points to final cluster centroids

Suppose that N denotes the total number of points, P represents the number of populations, T is the time of iteration, and D is the dimension of the dataset. The time complexity of PFA-GMM mainly depends on the following parts: (1) Calculating the covariance matrix by EM algorithm at the initialization stage is $O\left(D^{3}*P\right)$; (2) calculating the fitness values by EM algorithm during iteration is $O\left(T\left(P+D^{3}\right)\right)$; and (3) assigning data points to clusters takes $O\left(NlogN\right)$. Thus, the overall time complexity is $O\left(D^{3}*P+T\left(P+D^{3}\right)+NlogN\right)$.

## 5. Experimental Results and Analysis

In this section, we have validated the performance of PFA-GMM and compared it with other related clustering algorithms, including K-means [2], DBSCAN [28], GMM [23], Fuzzy C- means (FCM) [29], and Affinity Propagation (AP) [30]. To ensure a comprehensive comparison, we have also combined K-means with the Pathfinder algorithm. Additionally, we have adopted some metaheuristic algorithms such as Particle Swarm Optimization (PSO) [31], PSO-FCM [32], Genetic Algorithm (GA) [33], Artificial Bee Colony (ABC) [34], and Differential Evolution (DE) [35]. The parameters for the compared algorithms have been listed in Table 1. All datasets used in this section have been obtained from UCI datasets and synthetic datasets [36]. Furthermore, the numerical experiment to verify the effectiveness of the proposed algorithm includes the mean, standard deviation, and Wilcoxon rank-sum test [37] for fitness values. The iteration and population are set as 100 and 10, respectively. All the algorithms were coded in the Python programming language on Windows 10 with AMD Ryzen 5 2500U@2GHz and 8G RAM.

### 5.1. Clustering Evaluation Criteria

We have adopted four evaluation indices to test the performance of the clustering results, including Accuracy (ACC) [38], Normalized Mutual Information (NMI) [39], Rand Index (RI) [40], and Adjusted Rand Index (ARI) [41].

Accuracy (ACC): ACC means the proportion of correct labels to actual labels. The range of ACC is [0, 1]. When ACC equals 1, which tells that all the correct labels are found by the algorithm, the ACC is defined as follows:

(11) $$ACC=\frac{\sum_{i=1}^{K}x_{i}}{\left|N\right|}$$

where $x_{i}$ is a sample that is classified correctly, *K* is the number of clusters, and *N* is the number of data points in the dataset.

Normalized Mutual Information (NMI): NMI is a measure of the mutual dependence between two random variables, which is expressed as a ratio of the Mutual Information score and the average entropy of the two variables. Mutual Information measures the amount of information obtained about one random variable by observing the other variable, while entropy quantifies the expected amount of information held in a random variable. The NMI is defined as follows:

(12) $$NMI\left(X,Y\right)=\frac{I\left(X,Y\right)}{\sqrt{H\left(X\right)H\left(Y\right)}}$$

where $I\left(X,Y\right)$ represents the mutual information of variable X and variable Y, $H\left(X\right)$ and $H\left(Y\right)$ are the information entropy of variable X and variable Y, respectively. The NMI score ranges between 0 and 1, where a score of 1 indicates perfect correlation and a score of 0 indicates no correlation.

Rand Index (RI): RI is a similarity measure used to compare two different clustering methods. RI combines two sources of information, object pairs put together and object pairs assigned to different clusters in both partitions.

(13) $$RI=\frac{a+b}{\left(\begin{array}{c} n\\ 2 \end{array}\right)}$$

RI can be viewed as a measure of binary classification accuracy over the pairs of elements in a set, where *a* is the number of pairs correctly labeled as belonging to the same subset, and *b* is the number of pairs correctly labeled as belonging to different subsets. $\left(\begin{array}{c} n\\ 2 \end{array}\right)$ is the number of unordered pairs in a set of n elements. RI gives a value between 0 and 1, where 0 indicates that two clustering methods do not agree on the clustering of any pair of elements, and 1 indicates that two clustering methods perfectly agree on the clustering of every pair of elements.

Adjusted Rand Index (ARI): The ARI is a corrected-for-chance version of the Rand Index. The ARI rescales the index, taking into account that random chance will cause some objects to occupy the same clusters, so the RI will never actually be zero. The ARI is an improvement over the RI because it considers the expected value of the RI for random clustering.

(14) $$ARI=\frac{RI-E\left(RI\right)}{1-E\left(RI\right)}$$

where *RI* is the Rand Index and E(RI) is the expected value of the Rand index when the partitions are made at random while keeping the same marginal clustering distributions.

### 5.2. Experiments on Synthetical Datasets

**Figure 1:**
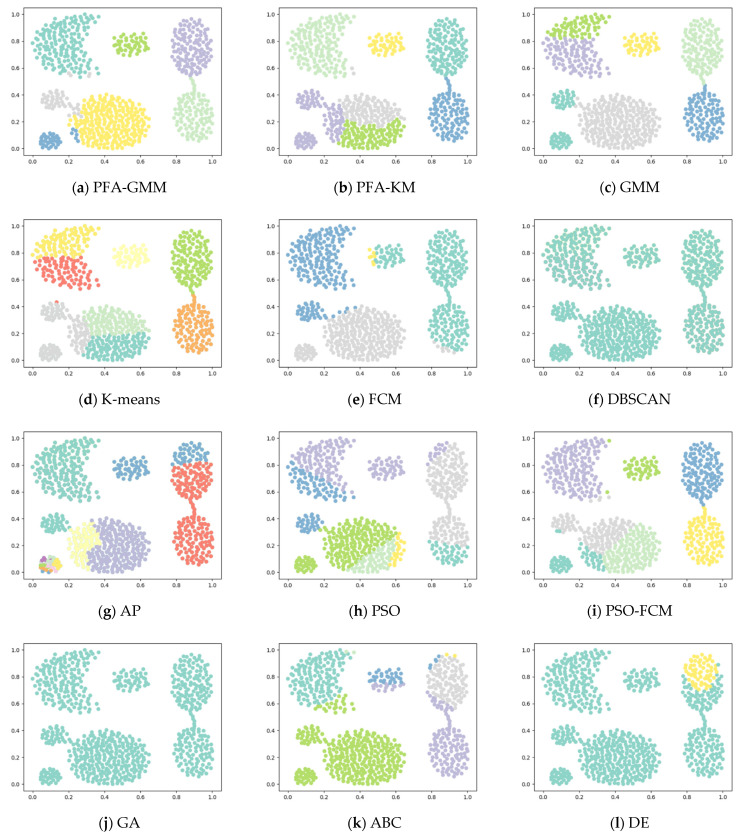
Clustering results on synthetic dataset-Aggregation.

**Figure 2:**
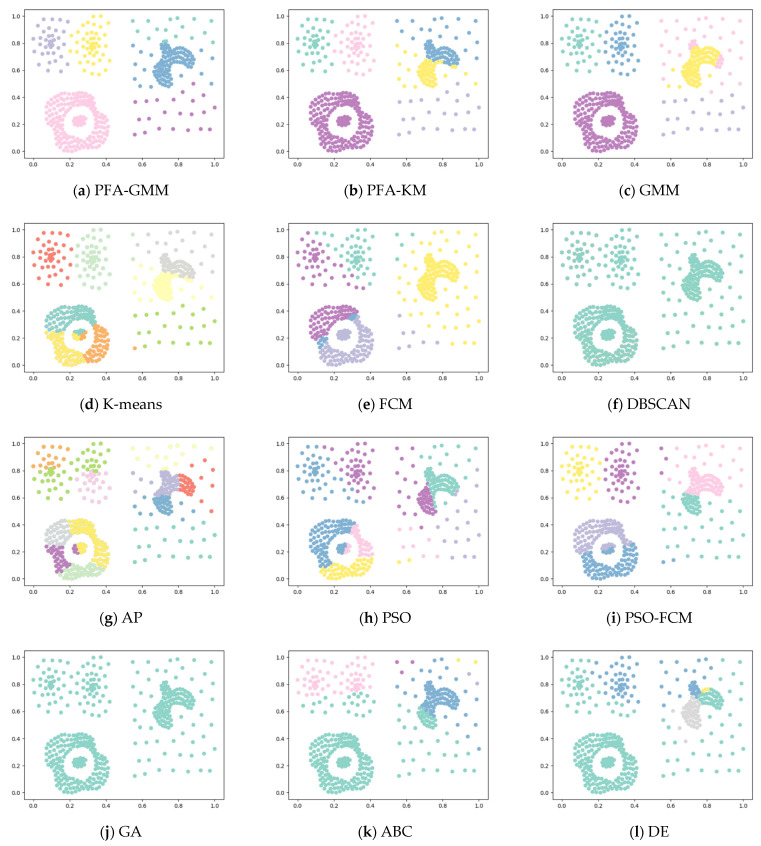
Clustering results for the eight methods on synthetic dataset-Compound.

**Figure 3:**
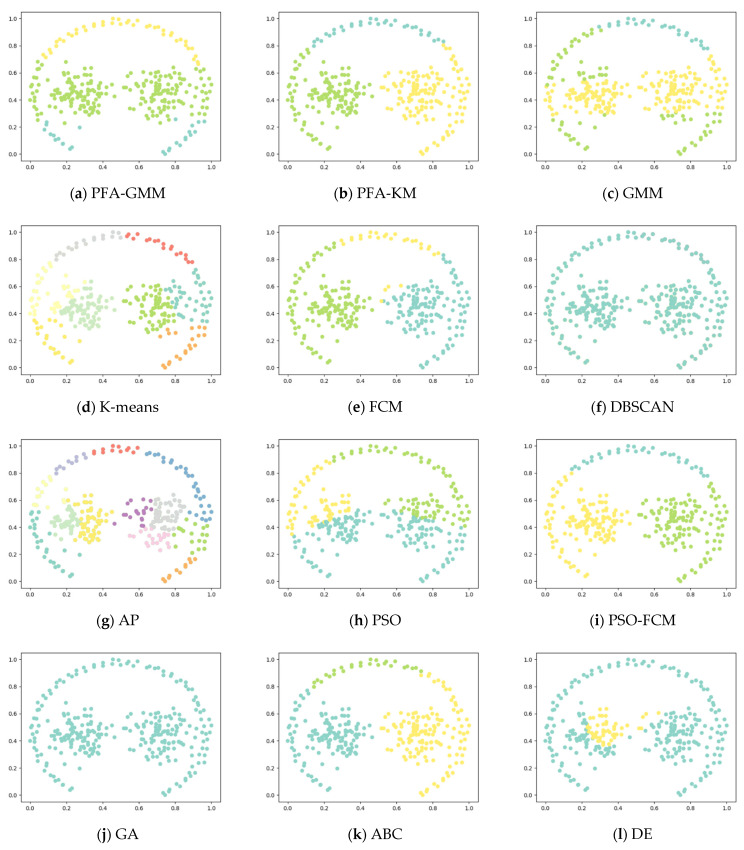
Clustering results for the eight methods on synthetic dataset-Pathbased.

**Figure 4:**
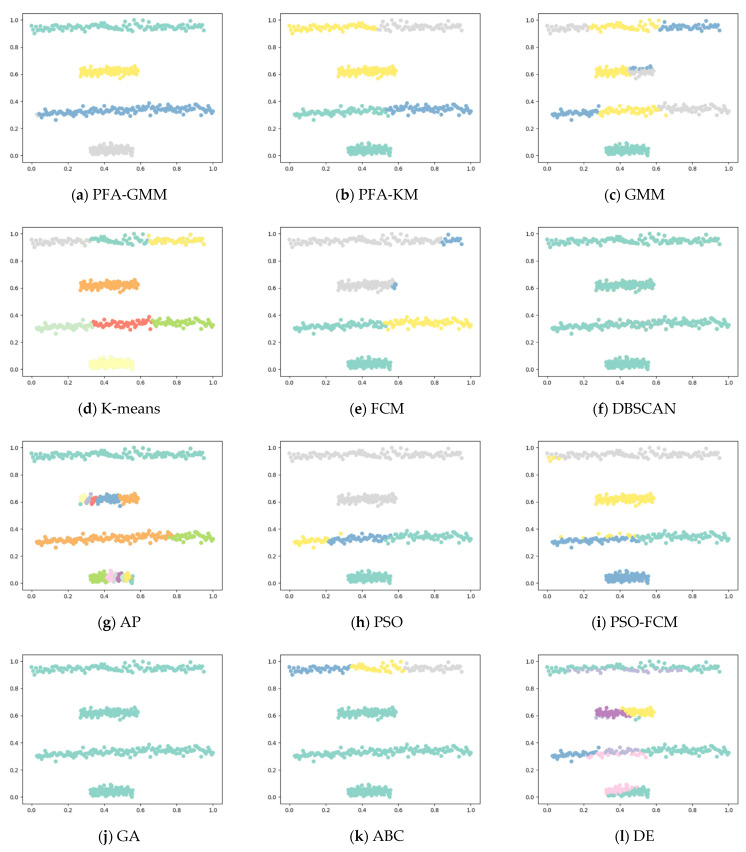
Clustering results for the eight methods on synthetic dataset-Four Lines.

**Figure 5:**
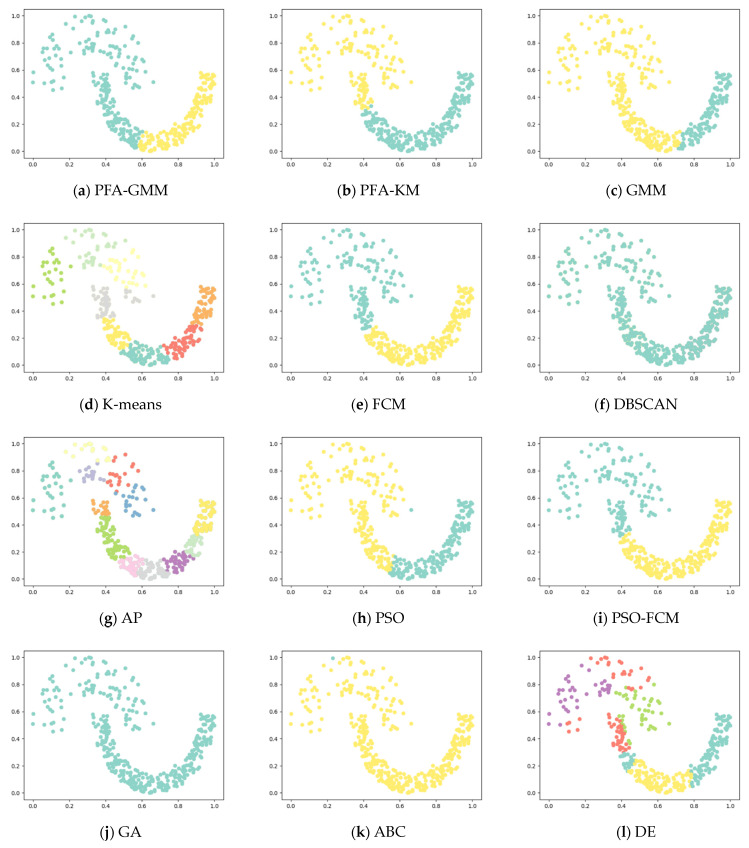
Clustering results for the eight methods on synthetic dataset-Jain.

**Figure 6:**
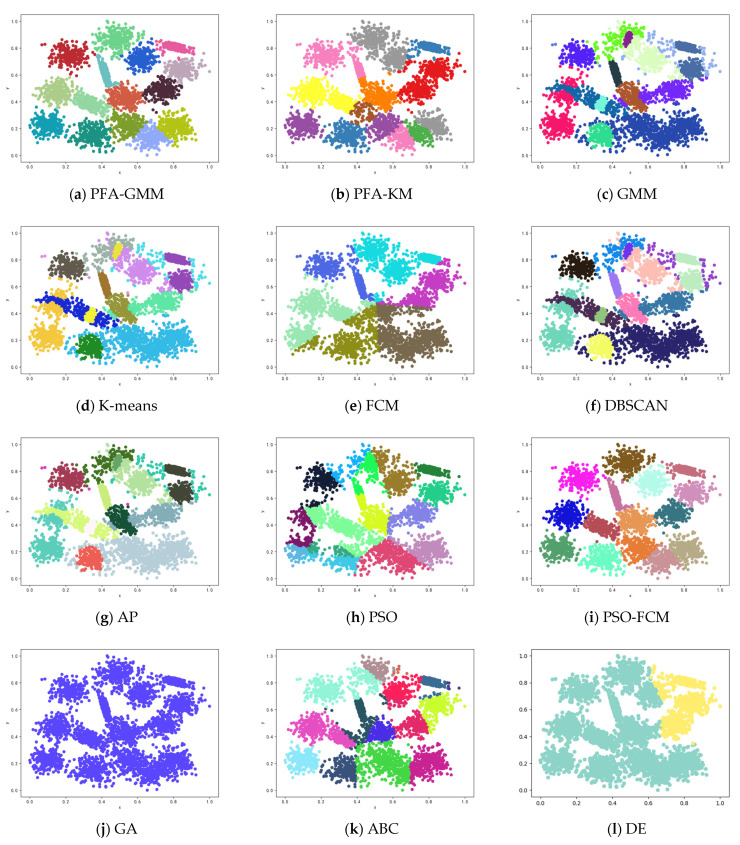
Clustering results for the eight methods on synthetic dataset-S2.

Six synthetic datasets are selected for variety (the features and instances of datasets are displayed below). The clustering result has been displayed as color figures in Figure 1, Figure 2, Figure 3, Figure 4, Figure 5 and Figure 6; we highlighted the best value for each dataset in bold. Table 2 shows the ARI, NMI, and ACC index values obtained by the proposed algorithm and other compared algorithms for synthetical datasets.

Table 2 shows that PFA-KM achieved superior results on Jain and Pathbased datasets, while PFA-GMM obtained the best results on S2, Compound, Four lines, and Aggregation datasets. Also the GMM algorithm leverages posterior probabilities for a soft assignment.

The clustering algorithms of K-means and its variants in the Aggregation dataset fail to consider the connectivity between clusters. K-means allocates data points to the nearest cluster center without regard for the boundaries between the points and the centers. As a result, PFA-GMM produces the most accurate clustering results among the algorithms tested. However, PFA-GMM still misallocates some boundary points to the wrong clusters.

In the S2 dataset, the PFA-GMM algorithm outperformed other algorithms. In contrast, the K-means algorithm failed to correctly differentiate data points of the close relationship between some clusters, which K-means identified as the same distribution. This issue led to a poor clustering result, which other algorithms also experienced since they shared the same problem as K-means regarding cluster differentiation.

In the Compound dataset, PFA-GMM obtained a better result than other algorithms, but it is not completely consistent with the actual distribution of the Compound dataset. However, most of the algorithms failed to differentiate clusters in the lower left corner of Figure 2.

In the Four Lines dataset, while K-means and its variants fail to accurately discriminate clusters due to their allocation of some data points to the wrong cluster centers based on the mean of data points, PFA-GMM results in the closest approximation to the actual distribution of the dataset.

In the Pathbased dataset, PFA-KM obtained a better result than other algorithms; most of the algorithms cannot assign data points correctly because this dataset contains manifold clusters, which most algorithms cannot differentiate accurately. K-means calculates the mean of data points and consider it as a proper candidate center that fails to recognize the cluster and allocates data points incorrectly.

In the Jain dataset, the clustering results of PFA-KM seem to closely resemble the actual distribution of the data. However, handling this dataset can be challenging due to the presence of non-convex clusters and closely related clusters. Most algorithms struggle to handle such datasets.

In order to evaluate the performance of our PFA-GMM algorithm, we not only employ ACC, ARI, and NMI index values but also use the Davies–Bouldin (DB) Index and Rand Index (RI) for comparing the performance with different algorithms. The Rand Index is a measure of the similarity between two algorithms on clustering. Both indexes range from 0 to 1, and the DB index value closer to 0 demonstrates better results. Table 3 shows the RI and DB index values obtained by the proposed algorithm and other compared algorithms for synthetical datasets.

### 5.3. Experiments on Real-World Datasets

Ten datasets from the UCI machine learning repository were used to evaluate algorithm performance. Each algorithm ran independently ten times, and the best value, worst value, mean, and standard deviation of fitness value were calculated and displayed in Table 4. Table 5 shows the Wilcoxon signed rank test for our proposed algorithm with other algorithms.

In this experiment, the results indicate that the optimal value of PFA-GMM on Glass, Liver, Wine, and Zoo datasets is superior to that of other algorithms. Similarly, the optimal value of ABC on Spect, Seeds, Iris, Breast, and Banknote datasets is better than that of other algorithms. Additionally, the GA algorithm outperforms other algorithms on the Heart dataset. On the other hand, the worst value of PFA-GMM on Glass, Heart, Liver, and Wine datasets is lower than that of other algorithms, while the worst value of ABC on Spect, Seeds, Iris, and Breast datasets is lower than other algorithms. In the case of PFA-KM, the worst value is lower than other algorithms on Zoo and Banknote datasets.

Furthermore, the mean of ABC on Spect, Seeds, Iris, Breast, and Banknote datasets is better than that of other algorithms. In contrast, the mean of PFA-GMM on Glass, Heart, Liver, and Wine datasets is better than other algorithms. Additionally, PFA-KM performs better than other algorithms in the Zoo dataset. In general, the mean of this data outperforms other algorithms.

The standard deviation of PFA-GMM on Spect, Glass, and Liver datasets is superior to that of other algorithms. Similarly, the standard deviation of ABC is better than other algorithms on the Seeds dataset, and the standard deviation of PSO-FCM is better than other algorithms on the Iris dataset. Lastly, the standard deviation of PFA-KM is better than other algorithms on the Breast, Heart, Wine, Zoo, and Banknote datasets.

A statistical method known as the Wilcoxon rank-sum test was used to analyze the fitness results from 10 independent runs. Table 5 shows the values generated by the test for pairwise comparison between PFA-GMM and other algorithms. The test compares the hypothesis that there is no significant difference between two values sampled from continuous distributions to the hypothesis that there is a significant difference. The results are statistically significant if the values in Table 5 are below 0.05.

## 6. Conclusions

In this paper, we introduce a novel clustering method by combining the Pathfinder algorithm with the GMM algorithm. The proposed algorithm aims to leverage the strengths of both algorithms to enhance the clustering performance. To evaluate the clustering performance, we compared our method, named PFA-GMM, with traditional clustering algorithms and swarm intelligence algorithms. We adopted ACC, NMI, and ARI clustering criteria to evaluate the performance of six synthetic datasets. Moreover, we calculated the fitness value for testing the performance of PFA-GMM on ten UCI datasets. Our proposed algorithm outperforms other algorithms on most datasets.

It is worth noting that PFA-GMM is based on an iterative algorithm, and therefore its running time on a large sample dataset will be relatively longer than that of traditional clustering methods. In future research, we will focus on improving the time complexity of this algorithm. Furthermore, we aim to enhance the accuracy of this algorithm on non-convex data and improve its performance on complex datasets. To achieve this, we plan to study allocation strategies to assign data points correctly and enhance their ability to cope with different shapes and density datasets.

## Figures and Tables

**Table 1:** Parameter values of compared algorithms.

Algorithm	Parameter	Value
PSO	$C_{1}$$,\;C_{2}$, Weight factor	0.5, 0.5, 1.2
PSO-FCM	$C_{1}$$,\;C_{2}$, Weight factor	2.0, 2.0, 0.4
GA	Crossover factor, Mutation factor	0.8, 0.01
ABC	Predetermined cycles	5
DE	Weight factor, Crossover probability	0.3, 0.8

**Table 2:** Clustering results of the algorithms on synthetic datasets.

DATASET	Algorithm	ARI	NMI	ACC	DATASET	Algorithm	ARI	NMI	ACC
**Aggregation** **(788 × 2)**	PFA-GMM	**0.97**	**0.93**	**0.94**	**S2** **(5000 × 2)**	PFA-GMM	**0.97**	**0.94**	**0.93**
	PFA-KM	0.75	0.84	0.69		PFA-KM	0.80	0.87	0.77
	GMM	0.87	0.93	0.91		GMM	0.66	0.79	0.56
	K-MEANS	0.65	0.81	0.61		K-MEANS	0.53	0.79	0.59
	FCM	0.73	0.73	0.67		FCM	0.40	0.68	0.42
	DBSCAN	0.35	0.00	0.00		DBSCAN	0.07	0.00	0.00
	AP	0.39	0.71	0.35		AP	0.41	0.69	0.44
	PSO	0.61	0.68	0.49		PSO	0.69	0.79	0.63
	PSO-FCM	0.83	0.84	0.72		PSO-FCM	0.97	0.94	0.93
	GA	0.35	0.00	0.00		GA	0.07	0.00	0.00
	ABC	0.82	0.75	0.67		ABC	0.76	0.82	0.67
	DE	0.42	0.16	0.05		DE	0.14	0.28	0.06
**Compound** **(399 × 2)**	PFA-GMM	**0.88**	**0.86**	**0.84**	**Patbased** **(300 × 2)**	PFA-GMM	0.47	0.31	0.14
	PFA-KM	0.78	0.80	0.77		PFA-KM	**0.75**	**0.55**	**0.47**
	GMM	0.86	0.85	0.83		GMM	0.45	0.21	0.13
	K-MEANS	0.55	0.68	0.46		K-MEANS	0.55	0.55	0.45
	FCM	0.61	0.60	0.44		FCM	0.70	0.48	0.42
	DBSCAN	0.40	0.00	0.00		DBSCAN	0.37	0.00	0.00
	AP	0.36	0.63	0.30		AP	0.28	0.52	0.24
	PSO	0.50	0.49	0.31		PSO	0.48	0.26	0.18
	PSO-FCM	0.66	0.70	0.54		PSO-FCM	0.75	0.55	0.47
	GA	0.40	0.00	0.00		GA	0.37	0.00	0.00
	ABC	0.65	0.48	0.42		ABC	0.72	0.53	0.45
	DE	0.59	0.33	0.26		DE	0.51	0.20	0.10
**Four Lines** **(511 × 2)**	PFA-GMM	**1.00**	**0.99**	**0.99**	**Jain** **(373 × 2)**	PFA-GMM	0.68	0.28	0.13
	PFA-KM	0.73	0.68	0.51		PFA-KM	**0.88**	**0.55**	**0.59**
	GMM	0.57	0.42	0.33		GMM	0.58	0.20	0.01
	K-MEANS	0.66	0.83	0.72		K-MEANS	0.28	0.40	0.17
	FCM	0.64	0.64	0.48		FCM	0.86	0.51	0.51
	DBSCAN	0.29	0.00	0.00		DBSCAN	0.74	0.00	0.00
	AP	0.24	0.52	0.18		AP	0.18	0.36	0.10
	PSO	0.57	0.65	0.47		PSO	0.72	0.29	0.19
	PSO-FCM	0.85	0.76	0.67		PSO-FCM	0.88	0.55	0.59
	GA	0.29	0.00	0.00		GA	0.74	0.00	0.00
	ABC	0.38	0.50	0.23		ABC	0.74	0.01	0.01
	DE	0.47	0.48	0.26		DE	0.42	0.38	0.26

**Table 3:** The DB index and RI results of each algorithm on synthetical datasets.

DATASET	Algorithm	DB	RI	DATASET	Algorithm	DB	RI
**Aggregation** **(788 × 2)**	PFA-GMM	**0.12**	**0.98**	**S2** **(5000 × 2)**	PFA-GMM	**0.08**	**0.94**
	PFA-KM	0.12	0.92		PFA-KM	0.09	0.94
	GMM	0.45	0.84		GMM	0.09	0.94
	K-MEANS	0.13	0.89		K-MEANS	0.11	0.93
	FCM	0.19	0.89		FCM	0.12	0.84
	DBSCAN	0.34	0.22		DBSCAN	0.47	0.07
	AP	0.17	0.84		AP	0.41	0.69
	PSO	0.15	0.89		PSO	0.09	0.94
	PSO-FCM	0.13	0.91		PSO-FCM	0.04	0.93
	GA	0.23	0.77		GA	0.36	0.07
	ABC	0.13	0.83		ABC	0.08	0.93
	DE	0.14	0.61		DE	0.08	0.34
**Compound** **(399 × 2)**	PFA-GMM	**0.14**	**0.92**	**Patbased** **(300 × 2)**	PFA-GMM	0.22	0.74
	PFA-KM	0.15	0.90		PFA-KM	**0.22**	**0.75**
	GMM	0.77	0.87		GMM	1.75	0.56
	K-MEANS	0.17	0.83		K-MEANS	0.23	0.74
	FCM	0.20	0.85		FCM	0.38	0.69
	DBSCAN	0.42	0.25		DBSCAN	0.49	0.33
	AP	0.17	0.80		AP	0.06	0.73
	PSO	0.22	0.73		PSO	0.22	0.72
	PSO-FCM	0.16	0.84		PSO-FCM	0.23	0.75
	GA	0.44	0.74		GA	0.35	0.58
	ABC	0.16	0.81		ABC	0.23	0.73
	DE	0.22	0.75		DE	0.36	0.59
**Four Lines** **(511 × 2)**	PFA-GMM	**0.07**	**1.0**	**Jain** **(373 × 2)**	PFA-GMM	0.48	0.57
	PFA-KM	0.19	0.83		PFA-KM	**0.39**	**0.79**
	GMM	0.27	1.0		GMM	0.53	0.51
	K-MEANS	0.07	0.91		K-MEANS	0.53	0.51
	FCM	0.30	0.75		FCM	0.81	0.59
	DBSCAN	0.54	0.25		DBSCAN	0.59	0.61
	AP	0.32	0.83		AP	0.65	0.46
	PSO	0.33	0.83		PSO	0.53	0.51
	PSO-FCM	0.35	0.82		PSO-FCM	0.39	0.78
	GA	0.45	0.59		GA	0.59	0.61
	ABC	0.46	0.57		ABC	0.58	0.62
	DE	0.38	0.62		DE	0.63	0.48

**Table 4:** Results obtained from 10 runs using real-world datasets.

Algorithm (Number × Feature)		Spect (267 × 22)	Seeds (210 × 7)	Iris (150 × 4)	Breast (699 × 9)	Glass (214 × 9)
**PFA-GMM**	Best	0.8058	0.3209	0.2033	0.5876	0.0512
	Worst	0.8058	0.4650	0.3456	1.0417	0.0809
	Mean	0.8058	0.3609	0.3010	0.6707	0.0603
	Std.	0.0000	0.0397	0.0433	0.1443	0.0087
**PFA-KM**	Best	1.1421	0.3268	0.2303	0.8663	0.2151
	Worst	1.1791	0.3297	0.3321	1.0950	0.4132
	Mean	1.1585	0.3288	0.2723	0.9406	0.2632
	Std.	0.0127	0.0011	0.0440	0.0998	0.0604
**PSO**	Best	1.0737	0.3286	0.2303	0.8069	0.2882
	Worst	2.1367	0.7367	0.7684	1.4967	0.4787
	Mean	1.4930	0.4889	0.4553	1.1616	0.3921
	Std.	0.2983	0.1318	0.1503	0.2063	0.0582
**PSO-FCM**	Best	1.1227	0.3317	0.3242	1.1236	0.3117
	Worst	1.4690	0.3363	0.3247	1.6832	0.3432
	Mean	1.2241	0.3332	0.3244	1.3712	0.3279
	Std.	0.1166	0.0017	0.0002	0.1896	0.0096
**GA**	Best	0.8218	0.2488	0.1902	0.5347	0.2022
	Worst	1.1003	0.3350	0.2529	1.0344	0.2815
	Mean	1.0089	0.2985	0.2136	0.7128	0.2527
	Std.	0.0957	0.0304	0.0221	0.1294	0.0225
**ABC**	Best	0.3079	0.1756	0.1798	0.1907	0.1531
	Worst	0.8413	0.3021	0.2303	0.6749	0.2628
	Mean	0.5714	0.2315	0.1996	0.3864	0.2095
	Std.	0.1727	0.0392	0.0176	0.1808	0.0384
**DE**	Best	1.1238	0.3412	0.2999	0.9923	0.2284
	Worst	1.6833	0.7104	0.6976	1.5695	0.5714
	Mean	1.3066	0.4789	0.4749	1.2934	0.4302
	Std.	0.1587	0.1161	0.1260	0.1534	0.0944
**Algorithm** **(Number × Feature)**		**Heart** **(303 × 13)**	**Liver** **(345 × 6)**	**Wine** **(178 × 13)**	**Zoo** **(101 × 16)**	**Banknote** **(1372 × 4)**
**PFA-GMM**	Best	0.2673	0.1176	0.1983	0.1230	0.3596
	Worst	0.4289	0.4183	0.2975	0.1742	0.6053
	Mean	0.2834	0.1927	0.2261	0.1571	0.5016
	Std.	0.0485	0.0794	0.0274	0.0137	0.1089
**PFA-KM**	Best	1.0394	0.1819	0.4875	0.1539	0.6004
	Worst	1.0394	0.5950	0.4937	0.1583	0.6004
	Mean	1.0394	0.2232	0.4913	0.1560	0.6004
	Std.	0.0000	0.1240	0.0029	0.0015	0.0000
**PSO**	Best	1.0871	0.7896	0.5331	0.1927	0.5152
	Worst	2.2302	1.7341	1.3147	0.3888	0.8781
	Mean	1.6378	1.0881	0.8028	0.2905	0.6749
	Std.	0.3839	0.2783	0.2102	0.0539	0.1150
**PSO-FCM**	Best	1.0908	0.7688	0.4701	0.1917	0.5998
	Worst	2.1732	0.8308	0.4801	0.3179	0.6064
	Mean	1.3155	0.7945	0.4752	0.2315	0.6023
	Std.	0.3145	0.0187	0.0026	0.0313	0.0020
**GA**	Best	0.1545	0.1306	0.4699	0.1833	0.2714
	Worst	1.1754	0.5463	0.4956	0.2547	0.4616
	Mean	0.9042	0.3455	0.4787	0.2250	0.3775
	Std.	0.3762	0.1507	0.0065	0.0212	0.0673
**ABC**	Best	0.2558	0.2558	0.2081	0.1523	0.1845
	Worst	0.8741	0.6353	0.4676	0.1983	0.6263
	Mean	0.5053	0.4117	0.3322	0.1668	0.2944
	Std.	0.1933	0.1418	0.1014	0.0137	0.1259
**DE**	Best	0.9603	1.0209	0.4824	0.2177	0.5461
	Worst	2.1271	1.8712	1.2315	0.4304	0.8467
	Mean	1.4722	1.3898	0.7852	0.3022	0.6600
	Std.	0.3907	0.3084	0.2398	0.0612	0.0993

**Table 5:** *p*-values produced by the Wilcoxon rank-sum test.

Algorithm	PFA-GMM vs.						
	PFA-GMM	PFA-KM	PSO	PSO-FCM	GA	ABC	DE
Spect	0.001953	0.001953	0.001953	0.001953	0.001953	0.009766	0.001953
Seeds	0.001953	0.003906	0.001953	0.037109	0.003906	0.001953	0.019531
Iris	0.001953	0.232422	0.001953	0.037109	0.009766	0.001953	0.005859
Breast	0.001953	0.001953	0.001953	0.001953	0.232422	0.019531	0.001953
Glass	0.001953	0.001953	0.001953	0.001953	0.001953	0.001953	0.001953
Heart	0.001953	0.001953	0.001953	0.001953	0.009766	0.064453	0.001953
Liver	0.001953	0.461451	0.001953	0.001953	0.037109	0.951915	0.001953
Wine	0.001953	0.001953	0.001953	0.001953	0.001953	0.027344	0.001953
Zoo	0.001953	0.556641	0.001953	0.001953	0.001953	0.431641	0.001953
Banknote	0.001953	0.232422	0.001953	0.232422	0.037109	0.037109	0.009766

## Data Availability

Not applicable.
